# Finding biomarkers of experience in animals

**DOI:** 10.1186/s40104-023-00989-z

**Published:** 2024-02-20

**Authors:** Sarah Babington, Alan J. Tilbrook, Shane K. Maloney, Jill N. Fernandes, Tamsyn M. Crowley, Luoyang Ding, Archa H. Fox, Song Zhang, Elise A. Kho, Daniel Cozzolino, Timothy J. Mahony, Dominique Blache

**Affiliations:** 1 School of Agriculture and Environment, The University of Western Australia, Crawley, WA 6009 Australia; 2 Centre for Animal Science, The Queensland Alliance for Agriculture and Food Innovation, The University of Queensland, St Lucia, QLD 4072 Australia; 3 School of Veterinary Science, The University of Queensland, Gatton, QLD 4343 Australia; 4 School of Human Sciences, The University of Western Australia, Crawley, WA 6009 Australia; 5 School of Medicine, Deakin University, Geelong, VIC 3217 Australia; 6 Poultry Hub Australia, University of New England, Armidale, NSW 2350 Australia; 7 College of Animal Science and Technology, Yangzhou University, Yangzhou, 225009 China; 8 Centre for Nutrition and Food Sciences, The Queensland Alliance for Agriculture and Food Innovation, The University of Queensland, St Lucia, QLD 4072 Australia

**Keywords:** Animal experience, Animal welfare, Biomarker, Stress, Welfare assessment

## Abstract

At a time when there is a growing public interest in animal welfare, it is critical to have objective means to assess the way that an animal experiences a situation. Objectivity is critical to ensure appropriate animal welfare outcomes. Existing behavioural, physiological, and neurobiological indicators that are used to assess animal welfare can verify the absence of extremely negative outcomes. But welfare is more than an absence of negative outcomes and an appropriate indicator should reflect the full spectrum of experience of an animal, from negative to positive. In this review, we draw from the knowledge of human biomedical science to propose a list of candidate biological markers (biomarkers) that should reflect the experiential state of non-human animals. The proposed biomarkers can be classified on their main function as endocrine, oxidative stress, non-coding molecular, and thermobiological markers. We also discuss practical challenges that must be addressed before any of these biomarkers can become useful to assess the experience of an animal in real-life.

## Introduction

The welfare of animals has been a concern to some segments of society for centuries [1, 2]. In Western societies, debate about animal welfare moved toward center stage from the middle of the last century and has continued to do so, while the issue became globally widespread in the 2000’s [3]. During these decades of debate, a recurrent premise has been that non-human animals (hereafter “animals”) experience the events in their life in a similar way to humans, and therefore people should treat animals with care and have respect for their welfare [1]. Along with the growing societal demand for better welfare for animals, a scientific approach to better understand and assess animal welfare has emerged. Progress in animal welfare science has laid the foundation for several frameworks that can be used to assess the welfare of animals that are under human care [1]. The nature of the experience that an animal has is central in all of these frameworks with diverse levels of importance [4]. Several behavioural and physiological indicators have been developed that are informative in the assessment of welfare [4]. For example, motor activity and body posture, cortisol, and heart rate can change in animals in response to a life event. Animal biologists have shown that these behavioural and physiological changes are controlled via modulation of the autonomic nervous system and the hypothalamic–pituitary–adrenal (HPA) axis [4]. While they are useful to assess the response of an animal to an experience, these indicators provide little information on the experiential process itself. In this review, we propose several biomarkers that may better reflect the experience of an animal during its lifetime. While most of the biomarkers that we identify are suitable primarily for mammals, further research may support their utility in other taxa. First, we briefly illustrate the use and limitations of existing indicators to assess animal experience. We then discuss several proposed biomarkers from the human biomedical sciences by outlining their relevance and their limitations to assess animal experience. After reflecting on the inherent difficulties of the validation of biomarkers of experience in animals, we offer some strategies to circumvent these difficulties. In conclusion, we describe the necessary features that a meaningful biomarker, or system of biomarkers, would require to estimate the experiential state of an animal.

## Where are we now?

At present, animal-based indicators can be divided into behavioural, physiological, and neurobiological indicators. They all provide valuable information on the mental state of an animal. The next section reviews biomarkers that are used currently and stresses how closely they are related to the experiential state. We deliberately focus mainly on the limitations that have been identified for each biomarker to demonstrate the potential gaps that new biomarkers could fill. The reader is encouraged to consult the cited references to appreciate the extent and the usefulness of the previous 60 years of work in this field in assessing animal welfare. We could not possibly cover that entire history in the present review.

### Behavioural indicators

Behavioural observation and several behavioural tests have been used extensively in the assessment of animal welfare [4]. Behavioural indicators are non-invasive, can be used on individual animals within groups, and are adaptable to many settings. Despite their practicality, the major limitations of behavioural indicators in the assessment of animal welfare are that they are either non-specific or too species-specific, affected by individual differences, and can be influenced by past and learned experiences (Table 1). Behaviours can be used as indicators of the experiential state of an animal on the assumption that certain behaviours are more likely to occur when an animal is in a positive or negative mental state [5]. However, behavioural changes are often indicative only of an extreme welfare state and mainly associated with a negative welfare state [6, 7]. Importantly, the behavioural indicators that are associated with a positive welfare are not very discriminative because they are usually essential behaviours, such as feeding and maternal behaviours [8, 9]. While behaviour can provide valuable information on how an animal is responding and coping in its environment, it does not necessarily provide insight into the experiential state of an animal.

**Table 1:** Example behavioral indicators of animal welfare, their uses, and limitations

Behavioral indicators and their uses	Examples	Limitations
Behavioural observations Certain behaviours are more likely to occur depending on whether an animal is in a positive or negative state [5]	Stereotypies (e.g., bar chewing in pigs and tongue rolling in cattle) [8, 10, 11] Aggressive behaviours (e.g., injurious feather pecking in poultry and tail biting in pigs) [12–15] Rebound activity, which is also used to indicate that an animal is motivated to perform that behaviour [16] Behavioural diversity, social behaviours (e.g., play) and maternal behaviours [17–19]	Focus generally on the presence of behaviours associated with negative mental states, such as abnormal or redirected behaviours Unreliable and non-specific (i.e., multiple mental states can produce the same behaviour) Influenced by level of arousal, which may be indicative of both positive and negative mental states Subjective and requires training/understanding of species-specific behaviours Influenced by past and learned experiences
Fear tests Measurement of the ability of an animal to cope in a challenging situation	Novel object, restraint, or isolation tests Used to assess fear in response and thus level of anxiety [20]	Focused solely on negative mental state The fear response is complex and varies at an individual level due to genetics and previous experiences [21] Can be contradictory in that animals may either respond actively (fight or flight) or passively (freezing) [21]
Cognitive bias tests (judgement, attention, and memory) The emotional state of an animal will impact the way they assess risks (judgement), choose what to focus on (attention), or remember (memory) an event	Animals in a negative mental state are thought to show negative bias in judgement, pay more attention to threats, and show impaired memory Higher frequencies of anticipatory behaviour in animals associated with more pessimistic judgements in cognitive bias tests [22]	Require animals to be trained to respond to cues associated with a rewarding or unrewarding event; influenced by previous experience; unreliable; and animals in poor welfare may show negative judgement bias, but the converse does not necessarily follow [23–28]
Preference and motivation tests Measurement of what and how much animals ‘want’ an environment or resource	Preference tests require animals to ‘work’ or pay a ‘cost’ to perform a behaviour or use a resource Motivation tests attempt to quantify the importance an animal places on a preference	Dependent on individual variation, environmental context and learned experience [29] Unreliable and non-specific (i.e., behaviour does not necessarily correlate to the actual experience of an animal)
Qualitative behavioural assessments Human assessment of an animal as a whole and how it interacts with its environment	Uses behavioural descriptors on a continuum ranging from low (e.g., calm, relaxed) and high (e.g., active, restless) levels of arousal Specifically validated in several species, including farm animals [30–40]	Subjective, context sensitive and relies on the ability of the human assessor [29] Influenced by past and learned experiences High arousal can be correlated with both positive and negative mental states, as can low arousal (e.g., indicating learned helplessness, freezing response, or calm)
Facial expression Facial expressions are reflective of emotional states	Facial expressions [41, 42] Eye white and movements, ear and body postures, movement patterns [43, 44]	Mainly been used in relation to negative mental states, for example use of facial expressions to measure pain Difficult to correlate specifically with the mental state of an animal Require species-specific data for accurate analysis due to species and breed variations
Vocalizations Vocalizations are expressed according to the experience lived by the animals	The use of vocalizations in animals as a form of welfare assessment have successfully been demonstrated in many farm animals including pigs, cattle, and horses [45–47]	Vocalization research in this field is measured under simulated situations where the animals are put in a setting that trigger vocalizations, resulting in ‘artificial’ vocalizations

### Physiological indicators

Physiological indicators of animal welfare (Table 2) have focused on the assessment of biological functioning rather than the experiential state of an animal [48–50]. The interpretation of any change in a physiological marker can be challenging because most physiological indicators respond to both positive and negative stimuli [51]. Variation in the method of measurement of a physiological response can hinder interpretation when samples derive from different sources, such as blood, saliva, hair, or feces [52]. The concentration of a hormone in different types of samples are not always correlated due to differences in time between synthesis, secretion, transport, metabolism, and action [48–50]. Moreover, it is difficult to establish the point where a physiological indicator reflects an adaptive response to a life event from the point where it reflects that an animal is no longer coping with that life event. Importantly, physiological indicators can reflect negative or neutral welfare states, but rarely a positive welfare state, which is a major limitation when trying to understand the full spectrum of experiential states of an animal.

**Table 2:** Example physiological indicators of animal welfare and their general response to stress

Physiological indicators	General response	Examples of stimuli that may generate a physiological stress response
Catecholamines (e.g., epinephrine and norepinephrine)	Increased in response to acute exposure to stressors	Increase observed in response to immune challenge (e.g., infection or disease), handling, feed restriction, transport, physical activity, and lameness [49]
Glucocorticoids (e.g., cortisol and corticosterone)	Increased in response to acute exposure to physiological or psychological stressors Normal/decreased in response to chronic exposure to physiological or psychological stressors	Increased cortisol concentrations in response to blood sampling, feeding, transport, handling, immune challenges, isolation and restraint, and exercise [50, 53–55]
Reproductive hormones (e.g., luteinizing hormone (LH), gonadotrophin-releasing hormone (GnRH))	LH concentration is suppressed during exposure to stressful events, can be increased by acute exposure to stressors and can be decreased during chronic exposure to stressors	Decreased or delayed LH surge in sheep and cattle in response to isolation, restraint, and transport [50] Decreased GnRH in sheep in response to electric stimulation administered short and long term and hypoglycemia [56] Increase risk of failing to ovulate or ovulate a low estrogenic follicle in dairy cattle with lameness [57]
Fatty acid intermediates (e.g., prostaglandins)	Increased during acute exposure to physiological stressors	Increased in lipogenic pathways while suppressing fatty acid oxidation in pigs in response to heat stress [58] Increased prostaglandin in rats in response to restraint and cage-switch stress [59, 60]
Metabolic enzyme markers (e.g., glucose, lactate dehydrogenase, creatine kinase)	Increased during acute exposure to physiological or psychological stressors	Increased gamma-glutamyl transferase in cattle in response to feed and housing changes [61]
Inflammatory markers (e.g., serum amyloid A, C-reactive protein)	Increased during acute exposure to physiological or psychological stressors	Increased acute phase protein, cytokines, and serum amyloid A concentrations in cattle in response to feed and housing changes [61] Increase in APP various species (including cats, dogs, poultry, horses, mice, sheep, pigs) in response to infectious and inflammatory disease, social stress, isolation, transport, tail biting, handling [62–65]
Immune system markers (e.g., total white blood cells, white blood cell type ratios, interleukins (Il-IB and IL-6), immunoglobulins (IgA), T-lymphocyte, cytokines)	Decreased during chronic exposure to physiological or psychological stressors	Decreased levels of IgA in piglets repeatedly confined in individual housing; rats exposed to an electric foot shock and psychological stress rats; and mice chronically restrained [66–68] Increased levels of IL-1 and IL-10 in mice with chronic-stress induced depression [69, 70]
Dehydroepiandrosterone (DHEA) and DHEA-sulfate (DHEA-S)	Increased during both acute and chronic exposure to stressors	Increased DHEA concentrations in horses with chronic stress; cattle in response to overstocking and transportation; and pigs after surgical stress [71–74] Increases DHEA:CORT ratios in pigs and cows in response to transport and novel environments [75]
Cardiovascular function (e.g., heart rate (HR), heart rate variability (HRV), blood pressure (BP))	Increased HR and decreased HRV during acute exposure to physiological or psychological stressors Increased BP during chronic exposure to physiological or psychological stressors	HR and HRV changes have been correlated with behaviour and positive and negative stimulus in horses, pigs, cattle, sheep, and dogs [76–81]
Respiratory function (e.g., respiratory rate (RR))	Increased RR during acute exposure to physiological or psychological stressors	Increased RR in sheep and cattle in response to heat stress [82–84] Increased RR in working dogs in response to physical activity after a competition [85]

### Neurobiological indicators

There have been several attempts to develop neurobiological indicators to assess animal welfare. Measuring the activity of systems of neural circuity and neurochemical reactions could offer a unique insight into the processes that are occurring in the brain of an animal [86–88]. As with behavioural and physiological indicators, neurobiological indicators have targeted mostly the negative experiential state, such as pain, fear, and stress, and to a lesser extent the circuits that are known to be associated with positive experience, such as anticipation and reward [29]. The activity of the neural circuits has been assessed by measuring specific neurochemicals (e.g., dopamine, serotonin, and endogenous opioids) in cerebrospinal fluid or neural tissue [89]. Neurochemical indicators are very valuable as they interrogate the communication networks with the brain, but they are not practical because the sampling techniques are invasive (e.g., to access cerebral spinal fluid or neural tissue) and the markers are often measured at a single time point. There are less invasive techniques that can be used to localize and measure the neurochemical and electrical activity of the brain, including neuroimaging techniques, such as functional magnetic resonance imaging, functional near infrared spectroscopy, and electroencephalography [90, 91], but these techniques also have limitations. While electroencephalography can measure brain activity in real time, the technique is not easily used in environments where most animals live, such as paddocks on a farm, and it cannot be used to assess many animals simultaneously. Beyond the research setting, the available neuroimaging techniques are impractical because they require specialized equipment and an animal needs to be restrained and sedated or anesthetized to be assessed, which can influence the results. Similarly, the available neurobiological indicators require extensive validation to become markers of the experiential state of an animal because levels of a mediator can be involved in different brain pathways and their activities are context dependent. For example, dopaminergic pathways are involved in several functions including pain, cognition, and personality, and can be modulated by several endogenous factors including oxidative stress [92, 93].

In the above sections, we have briefly argued that the available behavioural and physiological biomarkers, while informative, are proxies for the brain mechanisms that generate the experiential state. Neurobiological markers are a step closer to the neuronal activation that is linked to experience, but they are either too generic or impossible to measure in real-time or during real-life events. As pointed out in the introduction, an experiential state results from brain processes, so it seems obligatory to direct the search for relevant indicators using knowledge from the field of neuroscience [4].

## Where to go?

**Fig. 1:**
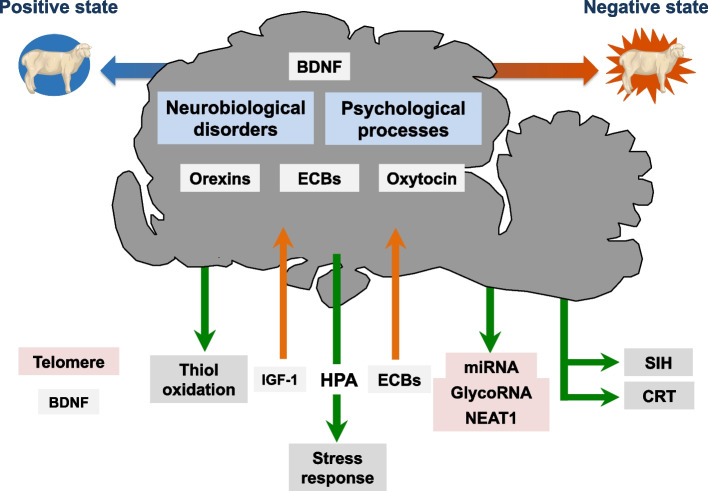
Schematic summary of potential biomarkers of the experiential state of an animal. These biomarkers are potentially involved in the shift to a more positive experiential state. The blue and red arrows represent the full spectrum of experiential state from negative (red end) to positive (blue end). The biomarkers were selected because they have been associated with neurobiological disorders (mood instability, anxiety, or depression) or psychological processes involved in emotion, temperament, or personality. Several of the candidate biomarkers of experience act in the central nervous system and are also present in the peripheral circulation, such as orexins, BDNF, oxytocin, IGF-1, and endocannabinoids. However, only peripheral levels of IGF-1 and endocannabinoids have been associated with changes in central processes that are linked to emotion or neurobiological disorders (illustrated by the orange arrows). MicroRNAs, glycoRNAs, and *NEAT1* could be efferent signals that are associated with experiential state and therefore could be measured in biological fluids such as serum, plasma, or saliva (green arrows). The experiential state of an animal could also affect its level of thiol oxidation, SIH, and CRT (green arrows) which could then be used as biomarkers (green arrows). The HPA axis and the stress response are not good biomarkers of experiential state because they are modulated by a large array of internal and external factors. Telomere attrition could be a long-time marker of experiential state, with a more positive experiential state resulting in less attrition. In addition of being produced in the brain tissue, BDNF is also produced in the peripheral tissue such as plasma and saliva. Abbreviations: BDNF: brain-derived neurotrophic factor, CRT: circadian rhythm of core body temperature, ECBs: endocannabinoids, HPA axis: hypothalamic–pituitary–adrenal axis, IGF-1: insulin-like growth factor 1, SIH: stress-induced hyperthermia

Concepts and methodologies from human neuroscience may offer some understanding of the experiential state of an animal [88]. In the following sections, we will discuss several candidate biomarkers (Fig. 1) by providing a short description of their biology and then a concise review of their association with either mental experience or mental dysfunction in humans. The selected biomarkers are either markers of cellular function or markers involved in the control of gene expression. The candidate biomarkers have been shortlisted based on their localization in areas of the brain that are involved in emotion, personality, and cognition and their involvement in psychiatric disorders such as mood and personality disorders, schizophrenia, anxiety, and stress-related disorders using first the literature in humans and additional information from animal models [89, 94]. We will briefly discuss the media in which the biomarkers can be found and the methods of detection. The list of proposed biomarkers is categorized according to the main function of each biomarker and comprises of endocrine, oxidative stress, non-coding molecular, and thermobiological markers.

### Endocrine makers

Each of the endocrine markers that is discussed below can be part of a complex control system and can affect downstream signals and/or effectors. We have chosen to target the main endocrine signals because the downstream effectors are often, if not always, under the influence of other endocrine or non-endocrine systems which might not react to the experiential state.

#### Oxytocin

Oxytocin is a nonapeptide hormone that is produced in the supraoptic and paraventricular nuclei of the hypothalamus. It is released by the posterior pituitary into the bloodstream from where it perfuses the body [95], and in the central nervous system (e.g., ventral tegmental area, frontal cortex, and brainstem) [96]. Oxytocin is known for its role in the contraction of smooth muscle that is associated with parturition and milk let-down, and its role in stimulating maternal behaviours in humans and other mammals [97]. Oxytocin receptors are found in several regions of the brain that are involved in the control of maternal behaviour, including the medial preoptic area, ventral tegmental area, and nucleus accumbens [97]. In humans, similar to other mammalian species, the cell bodies of neurons with oxytocin receptors are found in cortical regions and limbic structures, but not in the hippocampus [96]. A role for oxytocin in mood and personality disorders, schizophrenia, and autism has been established via studies that have investigated the plasma and CSF levels of oxytocin, mutations in the genes for oxytocin or oxytocin receptors, and responses to the administration of oxytocin [98]. Interestingly, in humans, comfortable physical contact increases the endogenous secretion of oxytocin [99]. Oxytocin has an apparent role during the expression of anxiety and stress because levels increase in those situations, but it might act as a circuit-breaker rather than as a direct correlate of the negative affective state. There is an interaction with the HPA axis [100, 101]. In response to an acute or a chronic psychological stressor, cortisol and oxytocin both increase in blood and saliva, but following that initial co-activation, during the recovery period indicated by changes in other stress makers, such as heart rate variability, oxytocin seems to have an anxiolytic effect and is associated with a reduction of cortisol synthesis [102–104]. Altogether, structural and functional studies in humans support a role for oxytocin in attenuating negative experiences such as anxiety and stress, as well as in eliciting positive experiences.

The intranasal or intracerebral administration of oxytocin attenuates the increase in cortisol concentration that occurs in response to various stressors, including social isolation, in humans and other animals [105, 106]. So, oxytocin seems to be related to positive life events. The serum concentration of oxytocin is correlated with positive social behaviours in cattle, rats, and primates, while there is no correlation between oxytocin and antagonistic behaviours [107]. The apparently anomalous involvement of oxytocin with the HPA axis could be an indication that oxytocin provides a measure of the stress resilience or coping capacity of an animal, rather than being a direct correlate of the stressor itself [101, 107–109].

Despite the apparent role of oxytocin in positive experience, validation is still necessary before oxytocin can be used as a reliable biomarker of the experiential state of an animal. It would be important to demonstrate a strong correlation between levels obtained centrally, in cerebrospinal fluid or by microdialysis in neural tissue, and peripherally in the blood (serum and plasma), urine, or saliva samples [95, 107, 109]. There may be some delay between the response observed centrally and levels in peripheral samples [109].

#### Growth hormone (GH), insulin-like growth factor one (IGF-1), and insulin-like growth factor binding proteins (IGFBPs)

Insulin-like growth factor one (IGF-1) is under the control of growth hormone (GH) and regulates growth and metabolism in the body [110]. To a large extent, the circulating levels of GH and IGF-1 depend on nutrient intake as well as the action of hormones that act on the hypothalamus, such as cortisol [111]. IGF-1 is produced in the liver, muscle, and fat and crosses the blood–brain barrier by active transport where it can impact on several brain functions, and is expressed in neurons and glial cells [110]. The paracrine and endocrine actions of IGF-1 in the central nervous system are modulated by insulin-like growth factor binding proteins (IGFBPs) which are also expressed in neurons and astrocytes [112].

IGF-1 is involved in general cellular growth, neural development, and neural protective mechanisms in the brain [110]. Dysregulation of the IGF-1 system has been associated with neurodegenerative diseases, such as Alzheimer’s, Parkinson’s, and Huntington’s disease [112]. In Parkinson’s disease, lower circulating levels of IGF-1 are associated with poor cognition [113], poor mood, and high anxiety [114]. Mice with knockout of both the insulin receptor and the IGF-1 receptor in the hippocampus and central amygdala present with anxiety-like disorders and impaired cognition, demonstrating that the IGF-1 signaling is involved in cognition and personality [115].

The GH/IGF-1 axis is affected by acute and chronic exposure to stressors. The expression of IGF-1 and IGFBPs is lower after exposure to stress, and both IGF-1 and IGFBPs respond dynamically to glucocorticoids, which could result in changes in cellular and neural functioning [110, 116, 117]. Overall, changes in the circulating levels of IGF-1 and possibly IGFBPs, could be correlated with changes in the experiential state in response to internal and external events [118]. Although their utility in that respect might be limited because they are secreted in pulsatile patterns and, as described above, they have complex interactions.

#### Brain-derived neurotrophic factor (BDNF)

Brain-derived neurotrophic factor (BDNF) is a very well-studied neurotrophin. BDNF plays an important role in the development and maintenance of brain function by mediating neuron survival and function. *BDNF* mRNA expression and BDNF immunoreactivity have been measured in the cortex, hippocampus, and amygdala of humans and rodents (for review see [119]). In humans, there is evidence for a role of BDNF in the pathophysiology of brain-associated illnesses. Patients with depression have lower expression of BDNF in brain tissue and lower levels in peripheral blood. Treatment of those patients with antidepressants can normalize those levels [120]. While the serum concentrations of BDNF are lower than normal in female patients with generalized anxiety disorder, the levels are not strongly related to anxiety disorders in general [120].

Similarly, in rodents, low levels of serum BDNF have been correlated with emotional and depressive-like behaviours [121, 122], and the level decreases following acute and chronic stress [123]. Like in humans, the administration of antidepressants increases the level of BDNF in the rat brain and the administration of BDNF into the hippocampus of rats produces antidepressant effects [124, 125]. BDNF, possibly by acting on synaptic plasticity, has been proposed to have a fundamental role in mediating changes in the central nervous system that are induced by experience and behavioural learning (for review see [126]). In rats, increases in the expression of *BDNF* mRNA and levels of BDNF in the medial forebrain, cerebral cortex, hippocampal formation, and hindbrain are associated with more play behaviour, improved spatial learning, and increased exploratory behaviour following environmental enrichment [127–130]. Importantly, increases in the level of *BDNF* mRNA in the hippocampus in response to a spatial memory test (Morris water maze) was not observed when rats were kept either in isolation or in a poor environment, suggesting that life situations that are perceived positive, such as enrichment or social contact, impact central BDNF [127–129]. Overall, evidence in humans and animal models suggests that the central expression of BDNF and potentially serum concentrations could be a biomarker of both the positive and the negative experiential state.

While the level of BDNF can be measured both centrally and peripherally, the usefulness of peripheral measures to assess the experiential state remains to be demonstrated. In pigs and rats, there was a moderate correlation between plasma and whole blood concentrations of BDNF and hippocampal BDNF (*r*^2^ = 0.41 to 0.44; [131]). Other studies have found no correlation between the peripheral and central level of BDNF, and to complicate matters further, the concentration of BDNF in saliva does not always reflect the concentration of BDNF in plasma [131–134]. The lack of correlation between the central and peripheral concentration of BDNF could be due to any of the multiple factors that are known to affect BDNF production such as sex, energy status and, as described above, the impact of life experiences. In addition to its expression in neural tissue, BDNF is produced centrally and peripherally in nonneuronal tissues [120], and at least in humans, the storage of BDNF in platelets contributes to up to a 200-fold difference between the concentration of BDNF in serum and plasma [135]. Importantly, different isoforms of BDNF have different biological functions, at least during neurodevelopment, that are influenced by serotonin [136]. The development of BDNF as a reliable marker of the experiential state will require demonstration of the biological relevance of the isoforms of BDNF and will also need to validate the type of sample, sampling technique, processing of samples, and storage that are all known to affect the peripheral concentration of BDNF [137, 138].

#### Orexins

Orexins, also known as hypocretins, are neuropeptides that are produced centrally [139]. The two orexins, orexin-A and orexin-B (also known as hypocretin-1 and hypocretin-2) are produced in neurons of the lateral hypothalamus, perifornical area, and dorsomedial hypothalamus [139]. Orexinergic neurons project throughout the central nervous system including the prefrontal cortex and other areas of the cortex and the amygdala [140].

Orexins play an important role in energy homeostasis, sleep, arousal, and reward processing [139]. In humans, lower concentration of orexins in either CSF or serum has been associated with a deficit in cognition and a higher incidence of neurodegenerative diseases such as Alzheimer’s, Huntington’s, and Parkinson’s disease [141]. A role for orexin in emotion and cognition has been suggested by studies that have applied one of the few antagonists and agonists of orexins [142]. In rodent models, orexins are involved in the modulation of mood, motivation, reward, and stress responses [143–145]. The central administration of orexins has an anti-depressant effect in mice and rats and thus supports a role for orexin in the modulation of mood and the coping capacity of an animal [146, 147]. In addition, the orexinergic pathways modulate several networks of neurotransmitters, including those that use dopamine, serotonin, and GABA, which are all involved in mental states including positive and negative emotions [142]. Recently, the reliability of the immunoassays that are used to measure orexins in serum, plasma, and CSF have been questioned, meaning that the established relationship between central and peripheral orexins will need to be validated with new analytics before they can be considered as a biomarker of experience in animals [148, 149].

#### Endocannabinoids

The endocannabinoid system includes the endocannabinoids, the enzyme that degrades them, and the endocannabinoid receptors [150]. Endocannabinoids are lipid-derived neurotransmitters, the most studied being N-arachidonylethanolamine (AEA) and 2-arachidonoylglycerol (2-AG). Both are synthesized in the peripheral and the central nervous system [151]. At the brain level, the endocannabinoids act via the cannabinoid receptor subtype 1 (CBR1) and those receptors are located mainly in regions of the brain involved in emotional behaviour, such as the prefrontal cortex in rodents [150]. The CBR1 receptor is expressed in GABAergic, dopaminergic, glutaminergic, and serotoninergic neurons in the medial prefrontal cortex [150]. Because the CBR1 receptor can be found in the neuron body, the axon, and the dendrites, the activity of the receptor can modulate the activity of those other neurotransmitters in several ways [150]. The endocannabinoid system has been implicated in the neuromodulation of several physiological systems, including pain [152], the stress response [153], anxiety [154], cognitive processing, and general homeostasis [155]. Interestingly, acute exposure to a stressor reduces the level of AEA in the synaptic space of neurons by increasing the activity of the enzyme that degrades AEA (i.e., fatty acid amide hydrolase) [153]. The endocannabinoid system is affected by stress and modulates the stress response [153].

A higher concentration of circulating endocannabinoids has been associated with reduced anxiety, less depressive behaviours, and elevated mood [156]. In rodents, studies using either antagonists of CBR1, CBR1 knockouts, or injection of endocannabinoids have demonstrated that the endocannabinoid pathway is involved in the behavioural expression of emotions, such as anxiety-like behaviours, during an elevated maze test [150]. While endocannabinoids have been measured in plasma and serum, they can also be measured in saliva, urine, milk, and hair [156]. In ruminants, the endocannabinoid system has been studied for its role in the control of reproduction [157–159], lipid metabolism and food intake [160], and the immune system [160]. However, to date no study has investigated the role of endocannabinoids in brain processes that are related to emotion reactivity. All of the data above suggests that endocannabinoids could be markers of the positive experiential state in animals, most probably linked to food intake and resilience to stress. The study of such associations will require well designed experiments because food intake can affect the level of circulating endocannabinoids by changing the activity of the microbiome [161].

### Markers of oxidative stress

Oxidative stress occurs when the homeostasis of oxidation–reduction activity is no longer maintained. A redox imbalance can be triggered by challenges that increase the production of reactive oxygen species (ROS) and reactive nitrogen species (RONS) and non-radical reactive derivatives (oxidants), or decrease the intake or synthesis of antioxidants, or increase the antioxidant turnover [162, 163]. Several types of challenge have been associated with an increase in oxidative stress including sepsis, mastitis, enteritis, pneumonia, metabolic disorders, and neurodegenerative disease [164]. The central nervous system is particularly sensitive to oxidative stress because of the high consumption of oxygen by neurons, the susceptibility of the lipid membrane of neurons to RONS, and the relative paucity of enzymes that reduce RONS [165]. RONS have been implicated in the neuronal death that is associated with the development of neurogenerative diseases, such as Parkinson’s and Alzheimer’s disease [166]. Interestingly, oxidative stress seems to interact with both IGF-1 and BDNF in the cognitive decline that is linked to neurodegeneration [166, 167]. In additional studies, 8-hydroxy-2-deoxyguanosine a marker of oxidative DNA damage, is used as a marker of cellular stress and supports a link between oxidative stress and depression. People with clinical depression have higher serum concentrations of 8-hydroxy-2-deoxyguanosine than controls [168, 169]. Long-term yoga practice had a positive impact on the mood state in people with clinical depression, but only tended to decrease levels of 8-hydroxy-2-deoxyguanosine in the urine [169]. The impact of oxidative stress on neurogenerative diseases seems to be related to a long-term imbalance in redox, suggesting that markers of oxidative stress could be useful indicators of long-term changes, rather than short-term changes, in the experiential state.

External environmental factors play a significant role in the prevention and mitigation of oxidative stress, mainly by increasing the level of antioxidants. In humans, nutritional antioxidants reduce the impact of neurogenerative and cognitive disorders [166]. In animals, in addition to dietary supplementation, the antioxidant status can be improved when environmental conditions induce a positive experiential state. Such environments include pasture-based systems for cattle, enriched housing systems for piglets, and protection from the cold for lambs [170–172]. Whether environmental enrichment causes the production of fewer oxidants, or improves antioxidant protection, remains unknown. Because of that association, the level of oxidative stress could serve to evaluate the positive experiential state of an animal. That could be achieved by assessing the redox balance by measuring reactive species and antioxidants [162, 164, 173].

The assessment of redox balance can be complicated because of the large number of biomarkers that are used to assess oxidative stress and antioxidants, their sensitivity to any metabolic change, and limitations of the detection technique [174]. Glutathione is a low molecular weight thiol-containing compound that is produced in the brain and has a neuroprotective role against oxidative stress via its role as a critical antioxidant [175]. Low levels of glutathione, centrally and peripherally, are indicative of oxidative stress, and those low levels correlate well with the severity of several neurodegenerative diseases and cognitive impairment [176]. Because thiol-redox homeostasis can have a significant role in neurodegenerative disease [175], the measurement of thiol-oxidation in biological media could be used to assess the experiential state. It is possible to measure the oxidation of cysteine in plasma albumin using the oximetric method [177]. The oximetric detection of thiol oxidation requires only a drop of blood and is highly sensitive. Measures of thiol oxidation are sensitive enough to detect changes in oxidative stress in response to exercise and muscle damage in humans and in response to a change in water quality in fish [178, 179]. The oximetric method, however, has not been validated in other species as a method of assessing oxidative stress.

### Non-coding molecular markers

Non-coding molecular markers, such as long noncoding RNAs (lncRNAs) and microRNAs (miRNAs), are found in many tissues including the brain. Non-coding RNAs can influence all types of cellular activity, from the general activity of neurons to complete cell apoptosis. LncRNAs and miRNAs could be relevant biomarkers of the experiential state since both have been linked to neuronal activity, neurodegenerative disease, and in some cases, associated with deficits in cognition [180].

In this section we discuss the potential of miRNAs, and one lncRNA, as biomarkers of experience. The lncRNA, the nuclear paraspeckle assembly transcript 1 (*NEAT1*), appears very promising as a biomarker of experience. GlycoRNAs, a modified RNA, will also be considered very briefly. Aside from the RNA-based biomarkers, we will also discuss the relevance of telomere length, since the attrition of telomeres has been linked to neurogenerative disease and cognitive decline [181, 182].

#### Micro-ribonucleic acid (miRNA)

MicroRNAs (miRNAs) are small noncoding RNAs (19–23 nucleotides) that influence gene expression by interacting with messenger RNAs (i.e., coding RNAs) [183]. MiRNAs play an important role in physiological and psychological functioning at a cellular and genetic level in higher organisms. MiRNAs, and other non-coding RNAs, also play a role in epigenetic changes that impact on gene expression and can be inherited, at least in the case of paternal stress (for review see [184]). In humans and other animals, the expression of miRNAs in the brain changes in response to changes in the external environment, such as sensory changes and dietary modifications, and has been associated with experiential states such as stress, depression and anxiety, and brain processes such as reward and decision making [183].

Negative mental states and psychological disorders have been associated with changes in miRNAs in brain tissue [185]. Changes in several miRNAs in the amygdala have been observed in mice that display anxiety- and depressive-like behaviours, as well as in mice that experience chronic social defeat [186, 187]. Acute and chronic stress causes alterations in the level of miRNAs in areas of the brain that are important in behaviour, emotion, and cognition [183]. In addition, changes in specific miRNAs are observed after the administration of antidepressant drugs (miR-16; [188]). MiRNAs have been implicated in the modulation of serotonergic (miR-135 and miR-16; [186, 189]) and dopaminergic reward pathways (miR-504; [189]).

In humans, circulating miRNAs have been proposed as informative biomarkers for mood disorders and as predictors of suicidal behaviour (for review see [190]). In rodents, miRNAs in the brain have been associated with human-like psychological disorders, but such associations have not been extensively studied in larger animals. Consequently, there is little information on the identification and annotation of miRNAs between humans and laboratory rodents, and other larger animals such as farm animals. The reference database for microRNA (release 22.1, accessed 16 July 2023, https://www.mirbase.org/) contains 2,654 mature miRNAs for humans, 1,998 for mice, but only has 1,025 mature miRNAs for cattle, 457 for pigs, and 10 for sheep [191]. It is unlikely that those numbers reflect differences in the biology of the species, but rather reflect the intensity of study. In addition, in farm animals, studies of miRNAs have focused mainly on miRNAs associated with production traits such as meat quality [192]. While miRNAs have been proposed to be potential biomarkers of animal welfare and health in livestock and poultry [193], most efforts toward the identification of specific miRNAs have focused on the ability of the animal to cope with external challenges and the associated stress response rather than experience [183].

Although the identification of miRNA biomarkers that might be associated with the experiential state of an animal remains a work in progress, the miRNAs offer a very promising avenue. Their measurement could be practical and highly specific. Even if they do not perfectly reflect brain activity, patterns of several miRNAs have been correlated between the blood and the brain suggesting that blood-based miRNAs can be used as a proxy for activity in the brain of certain miRNAs [194]. Further, the level of miRNAs in several biological fluids including blood, saliva, and urine, have been correlated [195], suggesting that it should be possible to assess the level of miRNAs in the brain with a non-invasive measurement method using easily accessible biological fluids, such as saliva. Moreover, recent advances in miRNA point-of-care technology will simplify the use of miRNAs as biomarkers [196]. To prove the utility of miRNAs as biomarkers of positive and negative experiences will demand several validation steps, not only to check the relevance of the miRNAs to the experiential status of animals, but also to confirm the robustness of the correlation between changes in the brain with changes in an accessible fluid for any miRNA of interest.

#### Nuclear paraspeckle assembly transcript 1 (NEAT1)

Nuclear paraspeckles are small cellular bodies that are found within the interchromatin space of the cell nucleus [197]. The structural core of a nuclear paraspeckle is nuclear paraspeckle assembly transcript 1 (*NEAT1*), a long noncoding RNA that binds together the other proteins that make up a paraspeckle [198–200]. As well as acting as a scaffold for the paraspeckle, *NEAT1* can alter the expression of many genes by impacting on the translation, transcription, and maturation of the microRNAs of those genes [197, 198]. *NEAT1* plays a role in cellular defence mechanisms by contributing to the maintenance of mitochondrion homeostasis [201]. *NEAT1* is important for neural development and functioning and has been associated with psychiatric diseases including Alzheimer’s, Huntington’s, and Parkinson’s disease [200, 202].

When *NEAT1* is knocked out in mice, and those mice are exposed to psychological stress during specific tests, such as the resident-intruder test and elevated plus maze test under bright light, they show abnormal behavioural responses including hyperlocomotion, an altered panic escape response, deficient social interactions, and impaired rhythmic patterns of activity [203]. These observations in *NEAT1* knockout mice suggest that changes in *NEAT1* could reflect the capacity of an animal to cope with psychological challenge and thus it may hold potential in the assessment of the associated mental state. Interestingly, the level of *NEAT1* increases in the peripheral blood of patients with Parkinson’s disease compared to non-affected patients [204] and could reflect the overexpression of *NEAT1* in the sustancia nigra of patients with Parkinson’s disease [205]. Levels of *NEAT1* in peripheral blood have been proposed as a biomarker of immune and liver diseases [206], and cancer [207]. It seems possible that levels of *NEAT1* in blood could serve as a biomarker of the experiential state.

#### Glycosylated ribonucleic acid (glycoRNAs)

GlycoRNAs were recently discovered in mammals and consist of RNA that has been modified with glycans that contain sialic acid [208, 209]. The majority of glycoRNAs are present on the cell surface and facilitate interactions such as intercellular trafficking and signaling via interaction with cell surface receptors [209, 210]. Amongst the small non-coding RNAs, the Y-RNAs (Y because they are found in the cytoplasm) are glycoRNAs [211]. Y-RNAs are essential for chromosomal DNA replication in vertebrates and are involved in the RNA stability and cellular functioning in response to stress [209, 212]. It has been proposed that glycoRNAs can modify cellular functioning and gene expression because they can interact with other cell surface molecules, such as the sialic acid binding-immunoglobulin lectin-type (Siglec) receptor [213]. In humans, genome wide association studies have identified variants in the gene encoding for Siglec receptors that are associated with neuropsychological conditions such as schizophrenia, bipolar disorder, and autism [213]. Since glycoRNAs bind Siglec receptors they could also be associated with neuropsychological disorders in addition to their involvement in the response to cellular stress. It could therefore be suggested that changes in the circulating level of glycoRNAs could reflect changes in the experiential state, most probably the negative state.

#### Telomere attrition

Telomeres are repetitive sequences (e.g., TTAGGG) within the DNA sequence at the end of chromosomes. Telomeres protect the coding regions of DNA from damage during normal cellular division and replication, and over time they become progressively shorter through telomere attrition [214, 215]. The rate and distribution of telomere attrition depends on the tissue and varies over time but overall, the average telomere length is correlated between different tissues within an individual [216].

The length of a telomere and its rate of attrition could reflect the cumulative lifetime experience of an animal and could also impact on the capacity of the animal to respond to challenge [217]. When the length of telomeres in white blood cells were monitored over a long term, an increase in the rate of shortening was associated with higher levels of psychological stress such as depression, anxiety, and social isolation in humans, and environmental stress such as lameness and hot weather in cattle [218–221]. In humans, factors such as optimism, physical activity, and positive social relationships have been suggested to slow the rate of telomere attrition [222–225]. In addition to causing DNA damage, telomere attrition may influence gene expression and therefore affect the capacity to generate a proper response after exposure to a stimulus. The influence of telomere attrition on gene expression could also impact brain functioning and therefore reflect the effect of cumulative exposure to stressors [226, 227]. A meta-analysis has concluded that telomere length is associated with neuropsychological conditions, with shorter telomeres reflecting the conditions [227]. Changes in telomere length and the rate of attrition are likely to be involved in persistent and long-term changes in positive and negative mental state.

Practically, telomeres can be measured in blood and tissue samples to assess relative telomere length at a single time point, or longitudinal sampling can be used to assess the rate of telomere attrition over time [226, 228]. When a method of measurement to assess telomere attrition is selected, and the results of tests are interpreted, factors such as cell type, animal age, and inter-individual differences need to be considered carefully [229].

### Thermobiological markers

#### Rhythmic changes in core body temperature

The core body temperature (T_c_) of endotherms, such as mammals and birds, varies in a circadian manner following a predictable pattern from day to night [230, 231]. For diurnal (awake and active during the day) endotherms, the core body temperature is typically higher during the day (active period) and is lower at night (rest period), creating a repetitive wave-like pattern over time. In contrast, the opposite is observed in nocturnal endotherms, with the temperature higher at night and lower during the day [232]. The pattern persists when an animal is deprived of environmental information about the time of day (such as light/dark cues or variation in environmental temperature), confirming that it is an endogenous circadian rhythm of T_c_ (CRT) [232]. The changes in T_c_ appear to be primarily under the control of the circadian system, which has input to the homeostatic thermoregulatory control system that activates thermoregulatory effectors [232].

A cosinor analysis of the CRT can be used to extract parameters that summarize the characteristics of the rhythm: the mesor (Midline Estimating Statistic Of the Rhythm), the amplitude (difference between the mesor and the peak or trough), and the acrophase (time of the peak) [233]. The parameters of the CRT change predictably with various biological factors such as body size, age, sex, activity level, and environmental factors such as feed availability and ambient temperature, in addition to some variation between species and individuals [232–234]. Changes in the physiology and behaviour of an animal can affect the circadian rhythm of its T_c_ [232]. For example, in sheep, changes in feed intake induce changes in the amplitude of the circadian rhythm of T_c_, and in lions, rabbits, and rats, the mesor of the circadian rhythm of T_c_ is affected by pregnancy [235].

Importantly for the subject of this review, the endogenous circadian system not only coordinates bodily functions with each other and with the external environment, but also integrates the zeitgebers and the psychological state in humans and other animals [236]. Disorders of the circadian rhythm in sleep patterns, mainly phase shifts, have been linked to bipolar disorder and seasonal affective disorder [237, 238]. In humans, a phase delay in the CRT has been linked to Alzheimer’s disease [239], while a reduced amplitude of the CRT and irregular shape of the CRT has been observed during episodes of depression, but not altered during manic phases [240].

#### Stress-induced hyperthermia

Stress-induced hyperthermia (SIH) is an acute response of T_c_, involving a rapid and transient increase in T_c_ in response to psychological stress [241, 242]. The SIH response is usually transient with a rapid increase in T_c_ after exposure to a stressor which gradually returns to normal once the stressor is removed. The HPA axis and brain areas including the glutaminergic, GABAergic, dopaminergic, and serotonergic pathways are involved in increasing thermogenic activity, which contributes to the observed increase of T_c_ during SIH [242, 243]. In rodents, the response involves the activation of brown adipose tissue and its associated metabolic heat production, as well as peripheral vasoconstriction [242]. In larger animals, the heat storage that is required for the increase in T_c_ cannot be achieved by peripheral vasoconstriction and must involve an increase in metabolic heat production, but the source of that heat remains unknown [244]. During the response to some challenges, it is impossible to dissociate the role of the psychological stress, per se, from other activity that involves an increase in metabolic heat production, such as the increase of muscular activity during flight from a predator. However, some psychological stressors do not induce an increase in muscular activity, so the response is independent of exercise hyperthermia. For example, SIH has been observed in response to handling, shearing, restraint, and social defeat in animals [242, 245–249]. Anxiety-like and depressive-like behaviours have also been shown to activate a SIH response [249]. Repeat or chronic psychological stress associated with more depressive-like behaviours has been shown to lead to anticipatory or learned SIH, changes in the CRT (e.g., hyperthermic response during active hours), and an exaggerated SIH to novel stressors [242, 250, 251].

The CRT and the SIH response both are sensitive systems and the parameters of the rhythms (especially the amplitude) and the SIH (the size) can be affected by positive or negative cues. These systems could be used to assess the experience of an animal since they are modified during psychological disorders, as described above. Infrared thermography has some potential in the assessment of negative experiential states but the results are not always conclusive or consistent (for review see [252]). Amongst the different parts of the body, the thermal imaging of lacrimal caruncle seems to be the most promising [252]. Infrared thermography could become a reliable biomarker of experience, but the technology might only be useful in a setting where the animal and the IRT device can be in proximity to each other with minimal environmental interference [252].

While to be truly informative of the CRT and SIH, body temperature needs to be measured deep in the body (the “body core”), the circadian rhythm of skin temperature in humans can be affected by mood [253], suggesting that variation in skin temperature could provide information that might be informative of the experiential state. There is a need to develop smart technology before the CRT or SIH can be used to assess experience in animals. However, changes in the CRT and the SIH response are still good research tools since experimental animals can be instrumented to collect the required data without any need to handle an animal during an experiment.

## Challenges in the search for biomarkers of the experiential state in animals

### Challenges during the identification phase

Any novel biomarker will need to be validated in a standardized experimental setting as well as controlled real-life situations before it can be used to assess animal welfare in a real-world setting. During experimental validation, any confounding variables that could interfere with the novel biomarker would need to be identified and controlled. For example, T_c_ can be affected by infection, exercise, and feed intake as well as the experiential state. Ideally, an experimental setting should, where possible, be relevant to in-field conditions to ensure that the results can be transferred from the experimental setting to the real-world setting [254]. It will be critical to validate any biomarker during both positive and negative experiential states. Except for the CRT, no other candidate described in this review seems to provide the capacity to vary in response to the entire range of experience. Therefore, it is likely that multiple biomarkers will be needed to assess changes in the experiential state of an animal.

Other challenges in the development of biomarkers will be the design of experimental paradigms that induce a range of experience and the establishment of thresholds for a negative, neutral, and positive experiential state. The range of experience, as perceived by an animal, will need to be based on other indicators, such as behavioural indicators, rather than a translation of the experience as perceived by a human to a given situation. It seems realistic to first adopt a categorical approach to the classification of experience (positive, neutral, and negative).

### Challenges during the implementation stage

For biomarkers to provide meaningful information on the complex dynamics of brain function over time, their measurement will need to be reliable, repeatable, and able to be collected from one of the biological matrices (blood, saliva, urine, etc.) using standardized protocols. Ideally, biomarkers of experience should be measurable when an animal is in its normal setting. The protocol to measure the biomarkers would require minimal or no disturbance to an animal, because if the procedure of sampling induces a change in the state of the animal, then the level of any biomarker will be confounded. For example, it would be preferable to measure biomarkers in easy-to-access matrices to avoid triggering any physiological responses to the sampling technique that may have an influence on the experiential state. Several techniques have been developed to measure biological samples in matrices other than blood and saliva, including in urine, feces, and hair. Moreover, the measurement of any biomarker of experience in a normal setting will require the techniques of sampling and analysis to be commercially feasible and practical for measuring both at an individual and group level [254, 255].

The case of glucocorticoids provides a good illustration of the need for the development of technology and careful sampling strategies. Glucocorticoids have been routinely measured to assess the response to stress and are commonly considered as an indicator of a negative welfare state. While glucocorticoids are often measured in plasma and serum in an experimental setting, it is commonly considered preferable to measure glucocorticoids or their metabolites in other biological matrices including saliva, urine, feces, and hair because these matrices can be sampled without the risk of inducing a glucocorticoid response [256–258]. The collection of a saliva sample can be achieved using some forms of environmental enrichment (for example toys), as has been done in pigs using cotton ropes to measure miRNAs in saliva for viral detection at a herd level [259] and chewable silicone sticks to measure saliva cortisol [256]. Saliva samples can also be used to measure other markers discussed in this review, such as the ratio of cortisol to DHEA in pigs and BDNF in rats [260, 261]. The measurement of glucocorticoids in each type of sample provides different information on the physiology of an animal due to differences in time between synthesis, transport, deposition in the matrix, and metabolism [49, 50, 54]. For example, the measurement of cortisol in saliva is often used to provide information on acute stress in response to an event, whereas hair samples capture information on chronic stress over weeks, months, or longer [257, 262].

There are many settings where it would be useful to measure biomarkers remotely with minimal need for interaction between humans and an animal. Techniques such as the bioacoustic analysis of vocalizations, analysis of facial expressions from video, or belts that transmit heart rate, although still limited in their application in the field, show promise for the automated assessment of animal welfare and perhaps the experiential state [41, 45, 46, 263, 264]. Technology is already available to remotely measure body temperature, heart rate, and locomotor activity in animals in the field, but these technologies remain expensive and rely on infrastructure to transfer data that could be used to assess the emotional state in real-time.

### Challenges to the practical use of biomarkers of mental state

In an ideal world, biomarkers of the experiential state of an animal could be used to develop a model that could predict the experiential state of an animal in a given situation. The development of such a model would involve the measurement of multiple indicators of brain function over short and long periods. The volume and variety of the data obtained from such an exercise would far outstrip the capacity of a simple analytical algorithm. To obtain a reliable and predictive model of experiential state from the complex dynamics of brain function, meaningful datasets must be acquired. To achieve meaningful datasets, repeated measurements should be taken from multiple replicates. Ideally, the samples that will form the training dataset of the model should be measured from experiments conducted across multiple facilities using a standardized protocol.

To be meaningful in a practical sense, the data on biomarkers of experiential state in animals must be relevant to the decisions that people need to make about the animals under their care. Models of biomarkers that reflect the experiential state of animals must provide the decision makers with actionable outputs that are compatible with the procedures and settings within which the animals are being kept. One strategy that has been trialed for the development of meaningful models of complex, real-world dynamics in other domains has been to involve non-scientist decision-makers and stakeholders in the modelling project [265]. Such a participatory modelling approach is thought to facilitate the integration of knowledge and ensure the ‘‘social robustness’’ of the outputs of the model and decision supports [266]. In the context of animal welfare, the term “stakeholders” goes beyond people with animals under their direct care to include anyone who is concerned about the welfare of the animals, including customers, auditors, regulators, and members of the public. Substantive approaches to participatory deliberation are based on the conviction that the quality of a decision is improved if such stakeholders are involved in the decision making process [267]. To ensure that information about the experience of animals is meaningful in practice, a collaborative approach should be used, such that the research process is designed and conducted with all stakeholders on equal footing understanding that there will be various degrees of participation [268].

Once a meaningful training dataset for biomarkers of the experiential state of an animal has been collected, the dataset should be pre-processed to remove any irrelevant data before a predictive model can be produced. In large and complex datasets, data pre-processing is the most critical stage in ensuring that only relevant information is retained without bias [269]. The cleaning step is challenging, as many factors, such as insufficient data, technical errors, and inaccuracies during data entry, can hinder the production of a clean and relevant dataset. Once that pre-processing is complete, then a predictive model can be produced using multivariate algorithms that use machine learning and/or deep learning techniques. The predictive model is produced by “learning” from the training sets that are acquired earlier in the process through either a supervised or an unsupervised approach. During supervised learning, the model is trained using a dataset that has been assigned with many observations, each containing several features and clinical outcomes for an animal. Conversely, an unsupervised learning model aims to identify patterns from the data without having access to classifications, such as a phenotype. As a final step, the model requires rigorous validation against an external dataset that was collected from an independent cohort of animals to address any potential selection bias. It is only after these time-consuming, and costly, steps are completed that the establishment of an accurate and predictive model can be achieved to make predictive and meaningful decisions about the experiential state of an animal.

## Conclusion

Although the behavioural, physiological, and neurobiological indicators that are currently available can provide valuable information on the welfare state of an animal, they do not provide the best assessment of the experiential state of that animal. We have proposed several candidate biomarkers that, based on their main function, include endocrine, oxidative stress, non-coding molecular, and thermobiological markers that may be correlates of the experiential state of an animal because they have been found to be affected by psychological or neurophysiological disorders in humans or in animal models of those same disorders. There is a need for further research to validate and improve our understanding of these biomarkers of experiential state and their dynamics. It seems unlikely that any single biomarker will cover the full spectrum of experience from negative to positive. Aside from validating these biomarkers, the assessment of experiential state will be most relevant to the assessment of animal welfare if the assessment can be conducted in real-time and anywhere. Therefore, further technological development is needed to facilitate the adoption of these novel biomarkers by the end users with animals under their care.

## Data Availability

None of the data was deposited in an official repository.

## Abbreviations

2-AG: 2-Arachidonoylglycerol
AEA: Arachidonylethanolamine
BDNF: Brain-derived neurotrophic factor
CBR1: Cannabinoid receptor subtype 1
CSF: Cerebrospinal fluid
CRT: Circadian rhythm of core body temperature
DNA: Deoxyribonucleic acid
ECBs: Endocannabinoids
FGF2: Fibroblast growth factor 2
GH: Growth hormone
GlycoRNA: Glycosylated ribonucleic acid
HPA axis: Hypothalamic–pituitary–adrenal axis
IGF-1: Insulin-like growth factor 1
IGFBPs: Insulin-like growth factor binding proteins
lncRNA: Long noncoding ribonucleic acid
miRNA: Micro-ribonucleic acid
mRNA: Messenger ribonucleic acid
NEAT1: Nuclear paraspeckle assembly transcript 1
ROS: Reactive oxygen species
RONS: Reactive nitrogen species
SIH: Stress-induced hyperthermia
T_c_: Core body temperature

## Glossary

Animal welfare: The transient state within an animal that relates to what the animal experiences, and mental state is the experience of an animal influenced by internal and external factors [270].
Animal experience or experiential state: Central processes integrate the perception of the environment with internal signals from bodily systems and cognitive inputs to generate a subjective state that includes, but is not limited to, sensations and emotional experiences. Animal experience changes according to changes in environmental and internal conditions. The quality of the experiential state ranges along a continuum from negative to positive experience. Experience varies between individuals according to their genetics, their history, and their perception of environmental cues.
